# Targeting NLRP3 Inflammasome in the Treatment of CNS Diseases

**DOI:** 10.3389/fnmol.2018.00320

**Published:** 2018-09-04

**Authors:** Bo-Zong Shao, Qi Cao, Chong Liu

**Affiliations:** Department of Pharmacology, Second Military Medical University, Shanghai, China

**Keywords:** NLRP3 inflammasome, CNS diseases, innate immunity, inhibitors, pharmacological application

## Abstract

Central nervous system (CNS) is one of the largest killers of people’s health all over the world. The overactivation of the immune and inflammatory responses is considered as an important factor, contributing to the pathogenesis and progression of CNS disorders. Among all kinds of immune and inflammatory reaction, the inflammasome, a complex of proteins, has been drawn increasingly attention to by researchers. The initiation and activation of the inflammasome is involved in the onset of various kinds of diseases. The NLRP3 inflammasome, the most studied member of the inflammasome, is closely associated with many kinds of CNS disorders. Here in this review, the roles of the NLRP3 inflammasome in the pathogenesis and progression of several well-known CNS diseases would be discussed, including cerebrovascular diseases, neurodegenerative diseases, multiple sclerosis, depression as well as other CNS disorders. In addition, several therapeutic strategies targeting on the NLRP3 inflammasome for the treatment of CNS disorders would be described in this review.

## Introduction

Innate immunity is a vital protectself-defensive mechanism against various kinds of internal and external threatening factors in organism (Thaiss et al., 2016; Leentjens et al., 2018; Suslov et al., 2018; Williams and O’Neill, 2018). The inflammasome is involved in the innate immune reaction, which, according to the name, is also defined as a special type of protein complex associated with inflammatory reaction, thus serving as connection between immune and inflammatory responses (Awad et al., 2018; Shibata, 2018). Among all kinds of inflammasomes, the NOD-like receptor family, pyrin domain containing 3 (NLRP3) inflammasome is the most studied one, which is involved in the pathogenesis and progression of various kinds of immune- and inflammation-related disorders, including central nervous system (CNS) diseases (Du et al., 2018; Gong et al., 2018; He et al., 2018; Sharma et al., 2018). So far, various kinds of therapeutic pathways taking advantage of the inhibition of the NLRP3 inflammasome have been developed or studied in the treatment of diseases based on our current knowledge on the NLRP3 inflammasome in diseases (Birnbaum et al., 2018; Mangan and Latz, 2018; McAllister et al., 2018; Singh and Jha, 2018). CNS diseases refer to a group of pathological processes occurring in CNS, leading to the damage of neural function or structure (Cacabelos et al., 2016). The overreaction of inflammatory and immune reaction has been considered as an important factor in the initiation and progression of various kinds of CNS diseases. Thus, targeting on the inhibition of inflammatory and immune reaction in CNS could contribute greatly to the alleviation of CNS diseases (Anuncibay-Soto et al., 2018; Rayasam et al., 2018; Sekerdag et al., 2018; Stephenson et al., 2018). Here in this review, we will discuss the roles of the NLRP3 inflammasome in several kinds of CNS disorders including cerebrovascular diseases, neurodegenerative diseases, multiple sclerosis, depression, and other CNS disorders such as traumatic brain injury. In addition, several therapeutic strategies against CNS diseases taking advantage of the NLRP3 inflammasome inhibition will be further discussed in this current review, aiming to illustrate a whole picture on the current knowledge and application of the NLRP3 inflammasome inhibitors in the treatment of CNS diseases.

## NLRP3 Inflammasome

The inflammasome, an inducer of innate immune reaction, functions in the recognition and targeting of numerous invasive or internal pathogens such as microbes (Malik and Kanneganti, 2017; Lugrin and Martinon, 2018). It is widely acknowledged that the inflammasomes are mainly produced in immune and inflammatory cells including macrophages, T lymphocytes and N&K cells, contributing to the triggering of the anti-pathogen immune inflammatory responses (Speeckaert et al., 2016; Correa-da-Silva et al., 2017; Di Virgilio et al., 2017). So far, several forms of inflammasomes have been described, mainly including the NLRP1, NLRP2, NLRP3, double-stranded DNA sensors absent in melanoma 2 (AIM2) as well as NLRC4 inflammasome (Ozaki et al., 2015; Duncan and Canna, 2018). Among them, the NLRP3 inflammasome is the most characterized and studied one, which has been demonstrated to be involved in the pathogenesis and progression of various kinds of diseases (Heo et al., 2018; Sano et al., 2018; Xiao et al., 2018; Zhai et al., 2018). Hereafter, the components and activation of the NLRP3 inflammasome will be discussed in the following contents.

**FIGURE 1:**
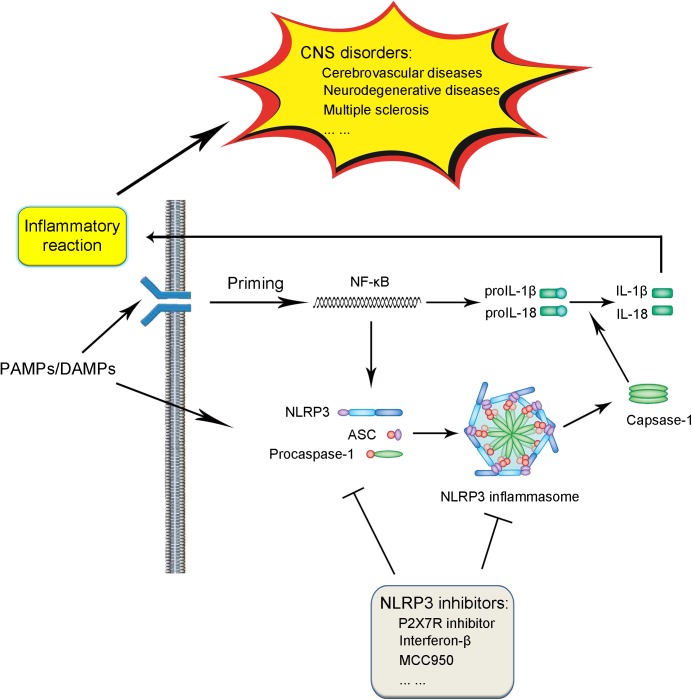
Schematic illustration of biological process and signaling pathway of NLRP3 inflammasome and association with CNS disorders. Under the exposure of PAMPs/DAMPs, NF-κB is activated, followed by the promotion of the NLRP3 protein as well as proIL-1β and proIL-18 production. The assembly and formation of the NLRP3 inflammasome through the combination of NLRP3, ASC, and procaspase-1 triggered by further stimulation leads to the production of caspase-1, which catalyzes the transformation from proIL-1β and proIL-18 into IL-1β and IL-18. The production and release of IL-1β and IL-18 induces various forms of inflammatory reaction, thus contributing to the pathogenesis and progression of CNS diseases. The process of NLRP3 inflammasome activation can be blocked by several kinds of NLRP3 inflammasome inhibitors, which serve as potential therapeutic strategies against CNS disorders.

The NLRP3 inflammasome is comprised of three components, including the NLRP3 protein, adapter protein apoptosis-associated speck-like protein (ASC), and procaspase-1 (Ito et al., 2015; Birnbaum et al., 2018). In the absence of activating factors such as pathogen-associated molecular patterns (PAMPs) and danger-associated molecular patterns (DAMPs), the leucine-rich repeats (LRRs) and NACHT domain in the NLRP3 protein connect with each other tightly to get rid of the interaction of NLRP3 protein and ASC (Shao et al., 2015; Kosmidou et al., 2018). Under the challenge of immune stimuli, the NLRP3 protein is activated, followed by the interaction with ASC and procaspase-1 on the pyrin domain (PYD) and caspase recruitment domain (CARD) in the NLRP3 protein, respectively, thus leading to the assembly of the NLRP3 inflammasome (Shao et al., 2015; Gambin et al., 2018; **Figure 1**).

Generally speaking, the activation of the NLRP3 inflammasome involves two steps (Sutterwala et al., 2014; Shao et al., 2015; Wu et al., 2018a; Yuan et al., 2018; **Figure 1**). In the first step, a priming signaling is triggered by certain PAMPs or DAMPs on Toll-like receptor 4 (TLR4), which leads to the activation of the NF-κB-mediated pathway. The triggering of NF-κB activation leads to the increase in the transcription of the NLRP3 inflammasome-related components, including the NLRP3 protein, pro-interleukin-1β (proIL-1β), and proIL-18. In the subsequent step two, with the further stimuli on immune and inflammatory cells, the NLPR3 protein was oligomerized, followed by the assembly of the NLRP3 protein, ASC and procaspase-1 into the complex of the NLRP3 inflammasome. The successful formation of the NLRP3 inflammasome triggers the transformation of the procaspase-1 to caspase-1, catalyzing the formation of the mature IL-1β and IL-18 from proIL-1β and proIL-18, which are secreted outside and lead to the cascade immune or inflammatory reaction (Place and Kanneganti, 2018; Shen et al., 2018).

So far, several factors have been demonstrated to lead to the activation of the NLRP3 inflammasome. For the initiation of the NLRP3 inflammasome, lipopolysaccharide (LPS) is widely considered to be a classic ligand for the activation of the TLR4 (Chu et al., 2018; Ho and Chang, 2018). In addition, several factors have been shown to be effective to induce the second step of the NLRP3 inflammasome activation, including the adenosine triphosphate (ATP, triggering the intracellular K^+^ efflux), PAMPs, DAMPs, silica, β-amyloid, autophagy deficiency as well as factors leading to the enhancement of mitochondrial Ca^2+^ overload and so on (Shao et al., 2017b; Chen et al., 2018; Li et al., 2018b; Meng et al., 2018; Zhao et al., 2018).

## NLRP3 Inflammasome in CNS Disorders

As we discussed above, the NLRP3 inflammasome is mainly produced in innate immunity cells, thus triggering the cascade immune and inflammatory reaction through the secretion of two pro-inflammatory cytokines including IL-1β and IL-18. As a result, the NLRP3 inflammasome is highly involved in the onset and development of various kinds of diseases, including cardiovascular diseases (myocardial ischemia/infarction, atherosclerosis, and hypertension), metabolic disorders (obesity, diabetes, and metabolic syndrome), digestive diseases (inflammatory bowel disease), renal diseases and CNS diseases (Chen et al., 2017; Kammoun et al., 2018; Martinez et al., 2018; Nasoohi et al., 2018; Sharma et al., 2018; Wu et al., 2018b; Yuan et al., 2018). In addition, it has been demonstrated that the polymorphism of the *NLRP3* genes may lead to the occurrence of some congenital disorders in patients and animal models (Lewis et al., 2011; Kuemmerle-Deschner et al., 2017; Landmann and Walker, 2017). For example, it was reported that the cryopyrin-associated periodic fever syndrome (CAPS), a group of rarely diagnosed hereditary autoimmune diseases including Muckle-Wells syndrome and familial cold auto-inflammatory diseases, was mainly led to by the gain-of-function mutations of *NLRP3* genes, which resulted in the overwhelming production and secretion of IL-1β and IL-18 (Kuemmerle-Deschner et al., 2017; Landmann and Walker, 2017). In CNS, it has been widely demonstrated that the NLRP3 inflammasome is highly involved in the pathogenesis and progression of various kinds of disorders, and strategies targeting on the inhibition of the NLRP3 inflammasome activation are increasingly developed and regarded as potential and effective pathways in the treatment of CNS diseases (de Rivero Vaccari et al., 2014; Zhou et al., 2016; Song et al., 2017). In this section, we will discuss the roles of the NLRP3 inflammasome in several well-known CNS disorders including cerebrovascular diseases (ischemic stroke and hemorrhagic stroke), neurodegenerative diseases (Alzheimer’s disease, Huntington’s disease, and Parkinson’s disease), multiple sclerosis, depression as well as other CNS disorders. Furthermore, the pharmacological applications of the NLRP3 inflammasome inhibitors in the treatment of CNS diseases are included in the relative parts of discussion (**Table 1**).

**Table 1:** Potential mechanisms of several NLRP3 inflammasome inhibitors in CNS diseases.

CNS disease	NLRP3 inflammasome inhibitor	Potential mechanisms	Reference
Ischemic stroke	Minocycline	Preventing microglia activation; Inhibiting Step 1 and 2 activation	Lu et al., 2016
	Sinomenin	Activating AMPK signaling	Qiu et al., 2016
	Nafamostat mesilate	Inhibiting NF-κB signaling	Li et al., 2016
Hemorrhagic stroke	P2X7R inhibitor	Inhibiting P2X7R	Feng et al., 2015
	Necrostatin-1	Inhibiting RIP1-RIP3-DRP1 signaling	Zhou et al., 2017
	MicroRNA-223	Downregulating NLRP3 expression	Yang et al., 2015
Alzheimer’s disease	Edaravone	Reducing mitochondria-derived ROS production; increasing SOD-2 activity	Wang et al., 2017
	Depeptidyl vinyl sulfone	Decreasing microRNA-155 and microRNA-146a	Falcao et al., 2017
Parkinson’s disease	MicroRNA-30e	Downregulating NLRP3, ASC and procaspase-1 expression	Li et al., 2018a
	Tenuigenin	Downregulating ROS	Fan et al., 2017
Huntington’s disease	P2X7R inhibitor	Inhibiting P2X7R	Sperlagh and Illes, 2014
Multiple sclerosis/EAE	Interferon-β	Inducting STAT1 phosphorylation and IL-10 production	Inoue et al., 2012; Inoue and Shinohara, 2013b; Shao et al., 2015; Inoue et al., 2016
	DRα1-mouse(m)MOG-35-55	Targeting on the MIF/CD74 pathway	Benedek et al., 2015, 2017
	HU-308	Activating CB2R; Inducing autophagy process	Shao et al., 2014
	PNU282987	Activating α7nAChR; Downregulating NLRP3 and β-arrestin-1 interaction	Ke et al., 2017
Depression	Salvianolic acide B	Promoting autophagy process	Jiang et al., 2017
	Astragaloside IV	Activating the PPARγ/NF-κB/NLRP3 inflammasome axis	Song et al., 2018
	Electro-acupuncture	Reversing the increase of P2X7R	Yue et al., 2018
Traumatic brain injury	MCC950	Suppressing the NLRP3 inflammasome priming process	Ismael et al., 2018
	Resveratrol	Promoting SIR1 signaling	Zou et al., 2018
	NOX2 inhibitor	Inhibiting NOX2	Ma et al., 2017

### Cerebrovascular Diseases

#### NLRP3 Inflammasome in Cerebrovascular Diseases

Cerebrovascular diseases refer to a group of pathological processes which result in the negative influence on cerebral vessels and circulation, thus leading to the subsequent damage of the brain tissues in function or structure. Cerebrovascular diseases are regarded as a group of major health problems for human beings worldwide, causing high morbidity, mortality as well as disability (Qureshi, 2004). It is widely acknowledged that there are two major types of cerebrovascular diseases, including ischemic and hemorrhagic stroke, with ischemic stroke referred to as decreased supply to certain brain regions and hemorrhagic stroke as bleeding into or around the brain tissue (Uchiyama, 2017; Gokcal et al., 2018).

The NLRP3 inflammasome has been reported to play a vital role in cerebrovascular diseases, and many signaling pathways mediated by the NLRP3 inflammasome are highly involved in the onset and progression of both ischemic and hemorrhagic stroke (Zhou et al., 2016; Barrington et al., 2017; Freeman et al., 2017; Ye et al., 2017; Fann et al., 2018; Nasoohi et al., 2018). For example, it was previously reported that under the stimulation of brain injury or disturbance of glucose metabolism in CNS, the activation of the NLRP3 inflammasome was triggered by thioredoxin-interacting protein (TXNIP), an endogenous inhibitor of the antioxidant thioredoxin (TRX) system, thus leading to the aggravation of brain tissue injury (Nasoohi et al., 2018). In addition, the purinergic 2X7 receptor (P2X7R)/NLRP3 pathway was shown to cause the enhancement of cerebral infarction volume and aggravation of neurological impairment through the triggering of the caspase-3 dependent neuronal apoptosis in cerebral ischemia models (Ye et al., 2017). Another toxin, the lysophosphatidylcholine (LPC) might trigger the activation of the NLRP3 and NLRC4 inflammasome in microglia and astrocytes, thus contributing greatly to the enhancement of neuroinflammation (Freeman et al., 2017). Furthermore, Fann et al. (2018) demonstrated that both the NF-κB and mitogen-activated protein kinase (MAPK) signaling pathways could serve as important signaling pathways in regulating the expression and activation of the NLRP3 as well as NLRP1 inflammasomes in primary cortical neurons and brain tissues under ischemic conditions.

#### Pharmacological Applications of NLRP3 Inflammasome Inhibitors in Cerebrovascular Diseases

As reviewed by us previously, many promising inhibitors of the NLRP3 inflammasome have been demonstrated in the treatment of inflammatory and immune diseases, such as Type I interferon and interferon-β, small-molecule inhibitors and others like autophagy inducers (Shao et al., 2015). In cerebrovascular diseases, several agents taking advantage of inhibiting the NLRP3 inflammasome have been presented in the alleviation of ischemic or hemorrhagic stroke. For the treatment of ischemic stroke, it was reported that pretreatment of minocycline, a tetracycline antibiotic, significantly prevented the activation of microglia through the inhibition of the NLRP3 inflammasome in the two steps of activation, thus improving neurological disorder, reducing infarct volume and alleviating cerebral edema (Lu et al., 2016). Additionally, a recent study demonstrated the neuroprotective effects of sinomenin, a kind of alkaloid derived from *Sinomenium acutum*, in mice cerebral artery occlusion (MCAO) ischemic models *in vivo* and oxygen glucose deprivation (OGD)-treated *in vitro* models (Qiu et al., 2016). Those protective effects were induced by the AMP-activated protein kinase (AMPK)-mediated inhibition of the NLRP3 inflammasome (Qiu et al., 2016). Furthermore, nafamostat mesilate, a wide-spectrum serine protease inhibitor, was shown to produce an anti-neuroinflammation effect in rat transient MCAO (tMCAO) mice as well as OGD *in vitro* models though the inhibition of the NF-κB-mediated activation of the NLRP3 inflammasome (Li et al., 2016). For the treatment of hemorrhagic stroke, it was previously demonstrated that blue brilliant G (BBG), a selective P2X7R inhibitor, contributed to the alleviation of the intracerebral hemorrhage-induced inflammatory injury in rat models through the inhibition of the NLRP3 inflammasome activation as well as attenuation of NADPH oxidase 2 (NOX2) and inducible nitric oxide synthase (iNOS) production (Feng et al., 2015). In addition, necrostatin-1 was reported to attenuate the early brain injury after subarachnoid hemorrhage in rat models through the inhibition of the receptor-interacting protein (RIP)1-RIP3-dynamin-related protein (DRP)1 signaling pathway, thus leading to the suppression of the NLRP3 inflammasome activation (Zhou et al., 2017). Moreover, it was shown that microRNA-223 could inhibit the activation of the NLRP3 inflammasome through the downregulation of the NLRP3 protein expression, thus alleviating the brain injury after intracerebral hemorrhage (Yang et al., 2015). Taken together, although current studies have provided potentially effective agents for the treatment of cerebrovascular diseases targeting on the NLRP3 inflammasome, further studies are demanded for the exploration of their applications in clinic.

### Neurodegenerative Diseases

#### NLRP3 Inflammasome in Neurodegenerative Diseases

Neurodegenerative diseases refer to a group of disorders with progressive loss of structure or function of neurons and finally lead to neuronal death (Haass and Selkoe, 2007). There are three well-known neurodegenerative diseases worldwide, namely Alzheimer’s, Parkinson’s, and Huntington’s diseases (Armstrong and Barker, 2001). One of the major causes of those three neurodegenerative diseases is regarded as the abnormal protein aggregation in neurons, including β-amyloid for the pathogenesis and progression of Alzheimer’s disease, α-synulein to form proteinaceous cytoplasmic Lewy bodies for Parkinson’s disease and the aggregate-prone huntingtin protein for Huntington’s disease (Chen and Tully, 2018; Clark et al., 2018; Kreutzer and Nowick, 2018; Matthes et al., 2018; Zhan et al., 2018; Zhang et al., 2018). Besides, the activation of the NLRP3 inflammasome also serves as a vital factor for the onset and development of neurodegenerative diseases (Ahmed et al., 2017; Fu et al., 2017; Li et al., 2017; Sarkar et al., 2017; Wu et al., 2017; Aminzadeh et al., 2018; Bai et al., 2018; Gong et al., 2018; Qi et al., 2018). It has been reported that the NLRP3 inflammasome activation significantly leads to the synaptic plasticity deficits in the pathogenesis of Alzheimer’s disease (Qi et al., 2018). Several influential factors have been reported to lead to the activation of the NLRP3 inflammasome, including cathepsin B, transient receptor potential melastatin 2 (TRPM2), mitochondrial impairment, microtubule-affinity regulating kinase 4 (MARK4), K^+^, Ca^2+^, and Cl^−^ fluxes and so on (Ahmed et al., 2017; Fu et al., 2017; Li et al., 2017; Sarkar et al., 2017; Wu et al., 2017; Aminzadeh et al., 2018; Bai et al., 2018; Gong et al., 2018). As a result, inhibiting the NLRP3 inflammasome may serve as a potential and effective therapeutic strategy in the treatment of neurodegenerative diseases.

#### Pharmacological Applications of NLRP3 Inflammasome Inhibitors in Neurodegenerative Diseases

In the last few years, numerous studies have been conducted in the exploration of therapeutic pathways against neurodegenerative diseases through the inhibition of the NLRP3 inflammasome. For the studies of Alzheimer’s disease, it was recently reported that edaravone, a recently shown oxidative stress suppressor, functioned in attenuating the β-amyloid-induced proinflammatory response in microglia (Wang et al., 2017). This effect was through the reduction of mitochondria-derived reactive oxygen species (ROS) production and increased manganese superoxide dismutase (SOD-2) activity, thus leading to the inhibition of the NLRP3 inflammasome-mediated proinflammatory secretion (Wang et al., 2017). Furthermore, depeptidyl vinyl sulfone, a chemical agent, was demonstrated to suppress the high-mobility group box protein-1 (HMGB1)/NLRP3 inflammasome-related inflammation in β-amyloid-stimulated microglia, which was involved in the decrease of the two inflammation-related microRNA including microRNA-155 and microRNA-146a (Falcao et al., 2017). For the treatment of Parkinson’s disease, it was reported that microRNA-30e might serve as a potential treatment for Parkinson’s disease therapeutics through the downregulation of the NLRP3 protein, ASC and procaspase-1 both in mRNA and protein levels, thus largely attenuating the NLRP3 inflammasome signaling (Li et al., 2018a). Furthermore, tenuigenin, a major active component derived from *Polygala tenuifolia*, was indicated to serve as a potential anti-inflammatory agent contributing to the treatment of Parkinson’s disease (Fan et al., 2017). The anti-inflammatory effect was induced by the inhibition of the NLRP3 inflammasome activation through downregulation of ROS in microglia in mice Parkinson’s disease models (Fan et al., 2017). For the treatment of Huntington’s disease, although not much studies are available for the exploration of the therapeutic roles targeting on the NLRP3 inflammasome, there is one study demonstrating that pharmacological inhibition of the ATP-sensitive homomeric P2X7R contributes to the alleviation of Huntington’s disease through the suppression of the NLRP3 inflammasome activation (Sperlagh and Illes, 2014). However, further efforts are demanded in the investigation of the NLRP3 inflammasome in the treatment of Huntington’s disease.

### Multiple Sclerosis

#### NLRP3 Inflammasome in Multiple Sclerosis

Multiple sclerosis is one of the most commonly diagnosed autoimmune diseases, characterized as demyelination and neurodegeneration, which also belongs to a neurodegenerative disorder (Shao et al., 2014; Rossi and Constantin, 2016). It is one of the most serious neurological diseases among young people, leading to progressive disabilities (Marck et al., 2016). The overreaction of the inflammatory and immune responses has been reported to contribute to the pathogenesis and progression of multiple sclerosis, in the occurrence of which was previously demonstrated to induce the highly expression of proinflammatory cytokines in microglia as well as CNS tissues in animal models of multiple sclerosis (Lieberknecht et al., 2017; Shao et al., 2017a; Lee et al., 2018a). The NLRP3 inflammasome has been reported to be involved in the development of multiple sclerosis through the secretion of IL-1β and IL-18 (Barclay and Shinohara, 2017). As a result, pharmacological inhibition of the NLRP3 inflammasome has been regarded as a potential target for the treatment of multiple sclerosis (Inoue and Shinohara, 2013a, 2015).

#### Pharmacological Applications of NLRP3 Inflammasome Inhibitors in Multiple Sclerosis

So far, several agents targeting on the inhibition of the NLRP3 inflammasome have been demonstrated to be effective in the treatment of multiple sclerosis or shown on the mice models of multiple sclerosis (experimental autoimmune encephalomyelitis, EAE). One of the most popular agents is interferon-β, which has already been applied in clinic as a first-line drug for the treatment of multiple sclerosis through the induction of the phosphorylation of STAT1 as well as IL-10 production (Inoue and Shinohara, 2013b; Shao et al., 2015). However, there were also studies demonstrating the limitation of the application of interferon-β in the treatment of the multiple sclerosis or EAE, showing that interferon-β therapy was effective against multiple sclerosis or EAE only in the NLRP3 inflammasome-dependent EAE (Inoue et al., 2012; Inoue et al., 2016). In addition, Benedek et al. (2015, 2017) demonstrated that DRα1-mouse(m)MOG-35-55, a less immunogenic alternative to two-domain class II construct developed by their lab, significantly reversed the clinical and histological symptoms of EAE mice through the inhibition of the NLRP3 inflammasome targeting on the MIF/CD74 pathway. Moreover, previous studies in our lab also reported several effective NLRP3 inflammasome inhibitors in the treatment of EAE. We found that the administration of cannabinoid receptor 2 (CB2R) agonist HU-308 significantly suppressed the activation of the NLRP3 inflammasome in microglia through the induction of autophagy process, thus producing an ameliorative effect on EAE (Shao et al., 2014). Furthermore, it was also reported by us that the activation of α7 nicotinic acetylcholine receptor (α7nAChR) by PNU282987 largely suppressed the NLRP3 inflammasome in monocyte/macrophage system in EAE, thus leading to the alleviation of the severity of EAE (Ke et al., 2017). Those effects were mainly through the downregulation of the interaction between the NLRP3 protein and β-arrestin-1 in microglia (Ke et al., 2017).

### Depression

#### NLRP3 Inflammasome in Depression

Another CNS disease highly related to the NLRP3 inflammasome is depression. Although it is regarded as a mental disorder, yet modern studies have demonstrated that the pathogenesis and progression of depression is involved in the overreaction of inflammatory and immune responses (Franklin et al., 2017). NLRP3-dependent caspase-1 activation was reported to be significantly implicated in the progression of systemic inflammation-induced depression triggered by LPS in mice depression model (Jeon et al., 2017). In addition, Lei et al. (2017) showed that neonatal inflammation or early-life inflammation stress could trigger the activation of the NLRP3 inflammasome through the up-regulation of the NLRP3 inflammasome-related proteins in the brain, thus leading to the increasing occurrence of anxiety-like behavior in adolescent rats. Consequently, targeting on the NLRP3 inflammasome might serve as a potential therapy in the alleviation of depression.

#### Pharmacological Applications of NLRP3 Inflammasome Inhibitors in Depression

So far, several agents have been reported to be effective in the alleviation of depression through the inhibition of the NLRP3 inflammasome. For example, it was recently demonstrated by Jiang et al. (2017) that salvianolic acid B, a natural compound extracted from *Salvia miltiorrhize*, contributed to the attenuation of depression in symptom through the promotion of the protective autophagy process, thus leading to the induction of the clearance of the NLRP3 protein. Astragaloside IV, an active component purified from Astragalus membranaceus (Fisch) Bge, attenuated the neuroinflammation-induced depressive-like behaviors in mice through the PPARγ/NF-κB/NLRP3 inflammasome axis (Song et al., 2018). In addition, other agents targeting on the inhibition of the NLRP3 inflammasome were reported in animal depression models, although the specific mechanisms remained unclear (Cao et al., 2017; Liu and Liu, 2017). Besides, it was recently reported that electro-acupuncture, one of the popular techniques of traditional Chinese medicine, contributed to the alleviation of the chronic unpredictable stress-induced depression and anxiety-like behaviors through the inhibition of the NLRP3 inflammasome activation, which was mediated by reversing the increase of P2X7R (Yue et al., 2018).

### Others

Besides those popular groups of CNS disorders discussed above, targeting on the inhibition of the NLRP3 inflammasome has been reported to be effective in other kinds of CNS diseases. For example, traumatic brain injury, a major cause of death and disability all over the world especially among children and teenagers, was considered to lead to the neuropathological conditions, which consequently cause the initiation of the production of the proinflammatory cytokines (Mortezaee et al., 2017). The NLRP3 inflammasome has been demonstrated to play a pivotal role in the development of traumatic brain injury through several kinds of mechanisms including RIP3-related pathway, activation of cortical microglia and so on (Lee et al., 2018b; Liu et al., 2018). As a result, inhibiting the NLRP3 inflammasome in CNS serves as a potential and effective pathway for the attenuation of the development of traumatic brain injury. For example, MCC950, a selective NLRP3 inflammasome inhibitor, was reported to alleviate the severity of traumatic brain injury in experimental animal models through the suppression of the NLRP3 inflammasome priming process (Ismael et al., 2018). In addition, resveratrol, a natural autophagy inducer, was demonstrated to function in the inhibition of the NLRP3 inflammasome in cerebral cortex through the promotion of sirtuin 1 (SIR1) signaling pathway (Zou et al., 2018). Furthermore, NOX2 was shown to contribute to the progression of traumatic brain injury, and the administration of the NOX2 inhibitors led to the a neuroprotective effect through the suppression of the NLRP3 inflammasome (Ma et al., 2017).

## Concluding Remarks

All in all, recent studies have demonstrated the important roles of the NLRP3 inflammasome in the pathogenesis and progression of various kinds of CNS diseases (**Figure 1**). So far, we are lucky to have many kinds of inhibitors of the NLRP3 inflammasome activation developed, which have already been shown to be effective in the alleviation of certain CNS diseases in patients or animal models. However, because of the limitations of modern studies, there is still a long way to go for their successful application in clinic and get rid of the side effects. Further efforts are demanded to develop potential and effective therapeutic strategies against CNS diseases taking advantage of the NLRP3 inflammasome inhibition.

## Author Contributions

B-ZS retrieved concerned literatures and wrote the manuscript. QC designed the table. CL revised the manuscript. All the authors agreed to be accountable for the content of the work.

## Conflict of Interest Statement

The authors declare that the research was conducted in the absence of any commercial or financial relationships that could be construed as a potential conflict of interest.
